# Emergence of Novel Type C Botulism Strain in Household Outbreak, Japan

**DOI:** 10.3201/eid2910.230433

**Published:** 2023-10

**Authors:** Rika Maeda, Misato Mori, Seiya Harada, Ichiro Izu, Takaaki Hirano, Yukie Inoue, Shunsuke Yahiro, Hiromi Koyama

**Affiliations:** Kumamoto Prefectural Institute of Public Health and Environmental Science, Uto, Japan (R. Maeda, M. Mori, S. Harada, I. Izu, T. Hirano, Y. Inoue, S. Yahiro);; Northern Kumamoto Administrative Headquarters Kamoto Area Development Bureau, Yamaga, Japan (H. Koyama)

**Keywords:** Botulism, *Clostridium botulinum*, botulinum neurotoxins, food poisoning, food safety, bacteria, Japan

## Abstract

In 2021, an outbreak of food poisoning caused by *Clostridium botulinum* type C occurred in Kumamoto, Japan. Analysis of the isolated strain revealed that it possessed the *bont/C* gene and was slightly different from the reference *bont/C* gene. The risk for human infection with this new toxin type may be low.

Botulism is a neuroparalytic disease caused by the botulinum toxin, which is produced by *Clostridium botulinum*. *C. botulinum* is physiologically divided into groups I–IV, and botulinum neurotoxins (BoNT) are classified into 7 types, BoNT/A–G. Human botulism is caused primarily by toxin types A, B, and E, and cases of human infection with *C. botulinum* group III, which produces toxin types C and D, are rare. Only 5 foodborne botulism outbreaks caused by *C. botulinum* group III (4 outbreaks caused by type C and 1 outbreak caused by type D) have been reported to date (*1*), and in Japan, only 1 infant botulism case caused by type C has been reported (*2*). *C. botulinum* group III is primarily known as an animal infection, and many of its toxin types have been reported as mosaic types (primarily in birds with toxin type CD and cattle with toxin type DC).

In 2021, foodborne botulism occurred in Kumamoto, Japan. A meal eaten in a domestic residence was the assumed cause, and 4 patients were affected. Botulinum toxin and *C. botulinum* were detected in 3 of the 4 specimens. A commercially prepared chicken dish was suspected to be the cause, but because no food was remaining, we were unable to conduct tests on it. We neutralized the toxin present in the specimens with type C botulinum antitoxin serum, and the isolated strain was found to carry the *bont/C* gene using PCR targeting the *bont* genes (*3*). Next-generation sequencing data revealed full-length coding regions of the *bont* gene of the isolated strains (GenBank accession no. LC759602). The next-generation sequencing method was as follows: after treating *C. botulinum* with 20 mg/mL lysozyme in 20 mM Tris-HCl, 2 mM EDTA, and 1% Triton X-100 (pH 8.0), we extracted DNA using a QIAamp DNA Mini Kit (QIAGEN, https://www.qiagen.com). We prepared genome-sequencing libraries using the QIAseq FX DNA Library Kit (QIAGEN) and sequenced the samples on the Illumina iSeq 100 (https://www.illumina.com). We analyzed sequencing data using CLC Genomics Workbench 22.0.2 (QIAGEN). The obtained contig was assembled from reads of 59× coverage and 29 kbp in size. In comparison with other known *bont* genes, the *bont* gene of the strain sequenced in this study had the highest amino acid sequence similarity with the *bont/C* gene (90%) but was partially different from the reference *bont/C* gene (Table). Detailed analysis revealed that the *bont* gene (LC759602) had the *bont/C* gene or the *bont/CD* gene in the protease domain (LC) and the translocation domain (H_N_), as well as the *bont/DC* gene in the receptor-binding domain (H_C_) (Table; Figure). The *bont* gene (LC759602) has not been previously reported, and we propose its designation as a new subtype of *C. botulinum* toxin.

**Table:** Amino acid percentage similarity between BoNTs (C, CD, DC, D, and LC759602) in study of novel type C botulism strain in household outbreak, Japan*

BoNT serotype (accession no.)	LC759602			
	BoNT gene	Protease domain	Translocation domain	Receptor-binding domain
BoNT/C(BAA14235)	89.79	97.73	93.57	78.01
BoNT/CD(BAA08418)	79.31	97.73	98.33	40.55
BoNT/DC(ABP48747)	72.87	48.05	74.45	98.57
BoNT/D(EES90380)	56.48	47.83	73.72	47.74

*BoNT, botulism neurotoxin.

**Figure:**
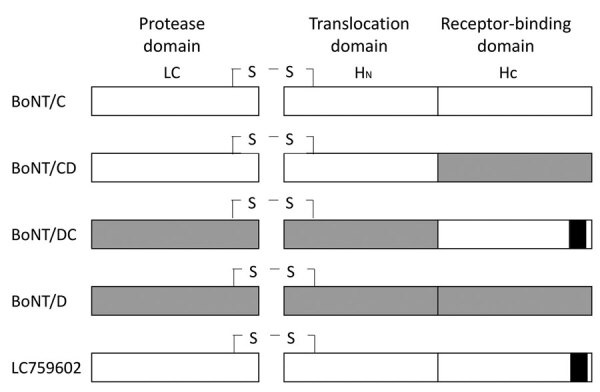
Schematic diagrams of each functional domain between BoNT (C, CD, DC, D, and LC759602) in study of novel type C botulism strain in household outbreak, Japan. Gray shaded areas indicate the partial sequence of the reference bont/D gene, white areas indicate the partial sequence of the cont/C gene, and black areas indicate the partial sequence of the reference bont/DC gene. BoNT, botulism neurotoxin.

The H_C_ domain is involved in neurotoxin binding to specific receptors in peripheral nerve terminals. The *bont* gene (LC759602) possesses the *bont/DC* gene in the H_C_ domain, suggesting that human susceptibility to this gene might differ from that of the reference BoNT/C toxin. Unlike other BoNTs, BoNT/C interacts only with gangliosides, and no protein receptor for this toxin has been identified (*4*). However, BoNT/DC has been reported to interact with gangliosides and protein receptors (synaptotagmin I and II) (*5*).

The *bont* gene (LC759602) was determined to be BoNT/C using PCR, which can easily distinguish between types C, D, CD, and DC of *C. botulinum* group III (*6*). It should be noted that, because not all type C strains were subjected to sequencing, the presence of the *bont* gene (LC759602) as type C, as determined by typing PCR, might already exist in other samples. Further investigation is needed to determine the proportion of *C. botulinum* carrying the *bont* gene reported in this study. The risk for human infection with this new toxin type should also be investigated in future research. However, given that human infections with a similar toxin type, *C. botulinum* group III, have rarely occurred, this new toxin type might pose little threat to human health.
